# Characterization of the Field Fludioxonil Resistance and Its Molecular Basis in *Botrytis cinerea* from Shanghai Province in China

**DOI:** 10.3390/microorganisms9020266

**Published:** 2021-01-28

**Authors:** Weizhen Wang, Yuan Fang, Muhammad Imran, Zhihong Hu, Sicong Zhang, Zhongqiao Huang, Xili Liu

**Affiliations:** 1 Department of Plant Pathology, College of Plant Protection, China Agricultural University, Beijing 100193, China; wzwangyx163@163.com (W.W.); fangyuan7852@163.com (Y.F.); imranpathologist@cau.edu.cn (M.I.); huzhihong@cau.edu.cn (Z.H.); 13379375613@163.com (S.Z.); huangzhongqiao@cau.edu.cn (Z.H.); 2 State Key Laboratory of Crop Stress Biology for Arid Areas, Northwest A&F University, Yangling 712110, China

**Keywords:** *Botrytis cinerea*, fludioxonil, fungicide resistance, biological characteristics, osmotic sensitivity, molecular mechanism, mutation

## Abstract

*Botrytis cinerea* is a destructive necrotrophic pathogen that can infect many plant species. The control of gray mold mainly relies on the application of fungicides, and the fungicide fludioxonil is widely used in China. However, the field fungicide resistance of *B. cinerea* to this compound is largely unknown. In this study, *B. cinerea* isolates were collected from different districts of Shanghai province in 2015–2017, and their sensitivity to fludioxonil was determined. A total of 65 out of 187 field isolates (34.76%) were found to be resistant to fludioxonil, with 36 (19.25%) showing high resistance and 29 (15.51%) showing moderate resistance. Most of these resistant isolates also showed resistance to iprodione, and some developed resistance to fungicides of other modes of action. *AtrB* gene expression, an indicator of MDR1 and MDR1h phenotypes, was not dramatically increased in the tested resistant isolates. Biological characteristics and osmotic sensitivity investigations showed that the fitness of resistant isolates was lower than that of sensitive ones. To investigate the molecular resistance mechanisms of *B. cinerea* to fludioxonil, the Bos1 amino acid sequences were compared between resistant and sensitive isolates. Resistant isolates revealed either no amino acid variations or the mutations I365S, I365N, Q369P/N373S, and N373S.

## 1. Introduction

*Botrytis cinerea* Pers.: Fr from the class Leotiomycetes and family Sclerotiniaceae is the causal agent of gray mold. It is a destructive necrotrophic plant pathogen that can cause serious losses in more than 200 crop species worldwide [1]. This fungus may not only cause rapid cell death on the aboveground parts of plants including the stem, leaf, flower, and fruit but also colonize plants systemically, without obvious symptoms in some cases [2,3]. The control of diseases caused by *B. cinerea* in agricultural production is still highly dependent on the application of fungicides. Due to the lack of registered compounds with multiple target sites, chemicals used for these diseases are mainly fungicides with specific modes of action that include succinate dehydrogenase inhibitors (SDHIs) such as boscalid and fluopyram; anilinopyrimidines (APs) such as cyprodinil, mepanipyrim, and pyrimethanil; ketoreductase inhibitors (KRIs) such as fenhexamid; quinone-outside inhibitors (QoIs) such as azoxystrobin, trifloxystrobin, and pyraclostrobin; and dicarboximides such as iprodione [4,5]. However, the fungicide resistance of *B. cinerea* to different compounds has been documented, resulting from point mutations at the target proteins or the overexpression of drug efflux transporters [6]. Moreover, *B. cinerea* is considered at high risk for fungicide resistance development because of its short lifecycle, prolific asexual reproduction capacity, and high genetic variability [7,8]. The frequent applications of fungicides in inappropriate ways make the fungicide resistance of *B. cinerea* a more widespread problem.

Fludioxonil, first introduced in 1993, belongs to the chemical class of phenylpyrroles, which are derived from the antibiotic pyrrolnitrin produced by different *Pseudomonas* species [9,10]. Fludioxonil has become one of the most important fungicides against grey mold and displays bioactivity against a broad spectrum of fungal pathogens [9,11]. This compound is toxic to *B. cinerea* by inhibiting its spore germination, germ tube elongation, and mycelium growth [9]. Even though its mode of action against fungi is not completely known, it is commonly believed that it could block the osmotic-regulatory signal transduction pathway [12,13]. The fungicide resistance of fludioxonil has been reported in different fungi, including *Neurospora crassa*, *Fusarium asiaticum*, *Alternaria brassicicola*, *B. cinerea*, and *Sclerotinia homoeocarpa*, and resistance is commonly conferred by point mutations in histidine kinase Os1 [13,14,15,16,17]. Thus, this protein has been considered as a primary target of fludioxonil in these organisms. Another well-documented mechanism of the resistance of *Botrytis* spp. to fludioxonil is associated with the overexpression of *atrB*, which encodes an ATP-binding cassette (ABC) transporter [18,19]. Determining the molecular basis of fungicide resistance in the field will be helpful for resistance monitoring and management to prolong the effective life of fungicides and safeguard their future use in crop protection [20].

Since the mid-1990s, fludioxonil has been widely used to control plant diseases caused by a variety of fungi including *B. cinerea*, but it has only been registered to control gray mold in China for several years [8,16,21]. The field fungicide resistance of *B. cinerea* to fludioxonil is still largely unknown in most places in China. In this study, *B. cinerea* isolates were collected from different districts of Shanghai province in 2015–2017. The main objectives were to (1) establish an overview of the resistance development of *B. cinerea* to fludioxonil in Shanghai province, (2) gain knowledge about the sensitivity of resistant isolates to other fungicides with different modes of action, (3) compare the biological fitness of resistant isolates with that of sensitive ones, and (4) reveal the molecular resistance mechanisms of these resistant isolates.

## 2. Materials and Methods

### 2.1. Collection, Isolation, Cultivation and Preservation of B. cinerea Isolates

Samples of diseased tomato leaves and fruits with typical gray mold symptoms were collected during 2015–2017 in fields of seven different districts (Baoshan, Chongming, Fengxian, Jinshan, the Pudong New Area, Qingpu, and Songjiang) in Shanghai province of China. For the isolation of *B. cinerea* from diseased leaves, small pieces of leaves were directly picked and transferred to potato dextrose agar (PDA) plates; for diseased fruits, conidia from fruit surfaces were gently dipped with sterilized cotton swabs and then transferred to PDA plates. Afterward, the PDA plates were placed at 17 °C in darkness. After three days of incubation, *B. cinerea* mycelia were obtained from the edge of the colonies and transferred to new PDA plates for further single-spore purification. For long-term storage, the cultures were transferred to PDA slants, covered with sterile mineral oil, and preserved at 14 °C in darkness.

### 2.2. Fungicides and Sensitivity Test

The fungicides and sensitivity test methods used in this study are listed in Table S1 and Table S2, respectively. All of the fungicides were dissolved in dimethyl sulfoxide (DMSO) to make stock solutions, and the fungicide stock solutions were diluted 1000 times with different media to the final working concentrations.

For fludioxonil sensitivity, a PDA medium was used, and the discriminatory concentrations of fludioxonil in the medium were set to 10 and 100 µg/mL, according to the results from a previous study by Zhou et al. [22]. Fresh mycelial plugs (5 mm in diameter) were taken from the growing edge of three-day-old *B. cinerea* colonies on the PDA and transferred to fludioxonil-amended plates. Each treatment was replicated three times. After incubation at 17 °C in the dark for three days, fungal growth was observed. The isolates that were able to grow at both concentrations were considered highly resistant (HR), those that could grow at 10 but not at 100 µg/mL were considered moderately resistant (MR), and those that could not grow at 10 µg/mL were designated sensitive (S). The following concentrations of fludioxonil were used to further determine the effective concentration inhibiting 50% of colony growth (EC_50_) values: 0, 0.01, 0.1, 0.5, 1, 10, 50, and 100 µg/mL.

For carbendazim sensitivity, fresh mycelial plugs (5 mm in diameter) were taken from the growing edge of three-day-old *B. cinerea* colonies on the PDA and transferred to yeast extract glucose (YG) medium plates amended with 100 µg/mL of carbendazim [23]. Each treatment was replicated three times. After incubation at 17 °C in the dark for three days, fungal growth was observed. The isolates that could grow at 100 µg/mL of carbendazim were designated resistant; otherwise, they were considered sensitive.

For azoxystrobin sensitivity, the discriminatory concentration of azoxystrobin in a yeast bacto acetate agar (YBA) medium was set to 100 µg/mL, and the medium was amended with 100 µg/mL of salicylhydroxamic acid to completely inhibit the alternative respiration pathway [24]. Fresh mycelial plugs (5 mm in diameter) were taken from the growing edge of three-day-old *B. cinerea* colonies on the PDA and transferred to the fungicide-amended plates. Each treatment was replicated three times. After incubation at 17 °C in the dark for three days, fungal growth was observed. The isolates that could grow at 100 µg/mL of azoxystrobin were considered resistant.

For SDHI fungicide sensitivity, the YBA medium was used, and the discriminatory concentrations of boscalid and fluopyram were both set to 10 µg/mL [25,26]. Fresh mycelial plugs (5 mm in diameter) were taken from the growing edge of three-day-old *B. cinerea* colonies on the PDA and transferred to the fungicide-amended plates. Each treatment was replicated three times. After incubation at 17 °C in the dark for three days, fungal growth was observed. The isolates that could grow at 10 µg/mL of these fungicides were designated resistant to boscalid or fluopyram; otherwise, they were considered sensitive.

For difenoconazole sensitivity, the YG medium was used, and the discriminatory concentration was set to 2.68 µg/mL (according to unpublished data), with DMSO as a control. Fresh mycelial plugs (5 mm in diameter) were taken from the growing edge of three-day-old *B. cinerea* colonies on the PDA and transferred to the fungicide- and DMSO-amended plates. Each treatment was replicated three times. After incubation at 17 °C in the dark for three days, the measurements were taken, and the inhibition efficiency of each isolate was calculated by comparing the colony diameters of the fungicide- and DMSO-amended plates. The isolates that could grow more than 50% under 2.68 µg/mL difenoconazole compared with DMSO were designated difenoconazole-resistant; otherwise, they were considered difenoconazole-sensitive.

For pyrimethanil sensitivity, a GKMN medium containing 10 g of glucose, 2 g of KH_2_PO4, 1.5 g of K_2_HPO_4_, 5 g of MgSO_4_·7H_2_O, 1 g of (NH_4_)_2_SO_4_, and 12.5 g of agar for 1 liter was used, and the discriminatory concentration of pyrimethanil in the medium was set to 1 µg/mL [25,27]. Fresh mycelial plugs (5 mm in diameter) were taken from the growing edge of three-day-old *B. cinerea* colonies on the PDA and transferred to the fungicide-amended plates. Each treatment was replicated three times. After incubation at 17 °C in the dark for three days, fungal growth was observed. The isolates that could grow at 1 µg/mL of the fungicide were designated to be pyrimethanil-resistant; otherwise, they were considered pyrimethanil-sensitive.

For cyprodinil sensitivity, the GKMN medium was used, and the discriminatory concentration of cyprodinil in the medium was set to 10 µg/mL [28]. Fresh mycelial plugs (5 mm in diameter) were taken from the growing edge of three-day-old *B. cinerea* colonies on the PDA and transferred to the fungicide-amended plates. Each treatment was replicated three times. After incubation at 17 °C in the dark for three days, the measurements were taken, and the inhibition efficiency of cyprodinil to each isolate was calculated by comparing the colony diameters of isolate on the fungicide- and DMSO-amended plates. The isolates that could grow more than 50% under 10 µg/mL of cyprodinil compared with those in DMSO were designated to be cyprodinil-resistant; otherwise, they were considered cyprodinil-sensitive [28].

For iprodione sensitivity, the PDA medium was used and the discriminatory concentration was set to 1 µg/mL [28], with DMSO as a control. Fresh mycelial plugs (5 mm in diameter) were taken from the edge of three-day-old *B. cinerea* colonies on the PDA and transferred to the fungicide- and DMSO-amended plates. Each treatment was replicated three times. After incubation at 17 °C in the dark for three days, the measurements were taken, and the inhibition efficiency of each isolate was calculated by comparing the colony diameters of the fungicide- and DMSO-amended plates. The isolates that could grow more than 50% under 1 µg/mL of iprodione compared with DMSO were designated to be iprodione-resistant; otherwise, they were considered iprodione-sensitive [28].

For gene expression level analysis, different isolates were incubated on the PDA medium at 17 °C in the dark for four days, after which the mycelia were collected and frozen at −80 °C until required. Total RNA was extracted from the frozen samples using the Eastep^®^ Super Total RNA Isolation Kit, and cDNA was synthesized using the abm^®^ 5X All-In-One RT MasterMix (with AccuRT Genomic DNA Removal Kit) according to the protocols of the manufacturers. Quantitative reverse transcription-PCR (qRT-PCR) was performed using a qTOWER 2.2 system (Analytik Jena AG, Jena, Germany). The transcript levels of the *atrB* gene were normalized against that of the reference genes encoding elongation factor 1 α and actin [29]. The 2^−ΔΔCt^ method was used to calculate the relative expression of the *atrB* gene [30]. This experiment was repeated twice.

### 2.3. Biological Characteristics of Sensitive and Resistant Isolates

For mycelium growth evaluation, *B. cinerea* isolates were transferred to PDA plates and incubated at 17 °C in darkness. After three days, mycelial plugs (5 mm in diameter) were excised with a sterilized hole punch from the edge of different colonies and placed upside down on new PDA plates of about 3 mm in thickness, with three replicates for each isolate. These plates were incubated at 17 °C in darkness for a further three days, and colony diameters were measured in two vertical dimensions.

For asexual reproduction capacity evaluation, *B. cinerea* isolates were first transferred to PDA plates for mycelial growth at 17 °C in darkness. Mycelial plugs (5 mm in diameter) were excised from the edge of three-day-old *B. cinerea* colonies and placed upside down on fresh carrot agar (CA) plates, with three replicates for each isolate. These CA plates were incubated at 20 °C in darkness for five days for mycelium growth before they were moved to 25 °C under black light (365 nm) for another five days. Then, 10 mL of distilled water were added into the plates to rinse the conidia. The conidial suspensions were filtered through four layers of muslin cloth, after which they were collected into 2-mL microtubes. The concentrations were counted with a hemocytometer under a microscope, and the sporulation capacity of each isolate was indicated as the number of conidia per cm^2^ of colony surface.

The conidial suspensions obtained from in vitro sporulation were spread over 1.5% water agar plates with a sterilized glass spreader for conidial germination. The water agar plates were incubated at 20 °C in darkness for conidial germination, with three replicates for each isolate. After 12 h, conidial germination was observed under a microscope. The conidium was considered germinated if the germ tube was longer than the conidium.

For asexual pathogenicity evaluation, *B. cinerea* infections were conducted using market-purchased tomato fruits of the same size. Tomato fruits were disinfected with 1% sodium hypochlorite for 1–1.5 min, rinsed three times with sterile distilled water, and left for several minutes to dry. Mycelial plugs taken from the edge of three-day-old *B. cinerea* colonies were placed upside down on the fruit surface that had been punctured with a sterilized needle, with three replicates for each isolate. The inoculated fruits were contained in sealed chambers with wet Whatman filter paper to retain sufficient moisture, and the chambers were stored at 20 °C with a 12-h light/12-h dark photoperiod. After three days, lesion area was measured on each fruit.

### 2.4. Osmotic Sensitivity Test of B. cinerea Isolates

For osmotic sensitivity, the PDA medium modified with a high concentration of NaCl (40 g/L) was used [22], with the normal PDA medium as a control. Fresh mycelial plugs (5 mm in diameter) were taken from the growing edge of three-day-old *B. cinerea* colonies on PDA and transferred to the NaCl- and non-amended PDA plates. Each treatment was replicated three times. After incubation at 17 °C in the dark for three days, the measurements were taken, and the inhibition ratio of NaCl was calculated by comparing the colony diameters of the NaCl- and non-amended plates.

### 2.5. Gene Amplification and Protein Sequence Alignment

Genomic DNA was extracted using the CTAB method adapted from a former study and stored at −20 °C until use [31]. Five primer pairs, BF1/BR1 to BF5/BR5, which were described in a previous study, were used for the amplification of the *Bos1* gene [32]. The final volume for PCR was 50 µL, containing 5 µL of a 10X Standard Taq Reaction Buffer, 4 µL of 10 mM dNTPs, 1 µL of a 10 µM Forward Primer, 1 µL of a 10 µM Reverse Primer, 0.5 µL of a Taq DNA Polymerase enzyme, 1 µL of template DNA, and 37.5 µL of nuclease-free water. The PCR reaction was performed with initial preheating for 3 min at 95 °C, followed by 40 cycles of denaturation at 94 °C for 1 min, annealing at 60 °C for 1 min, and extension at 72 °C for 1.5 min, and a final extension at 72 °C for 5 min. PCR products were sent for sequencing to Tsingke Biological Technology (Beijing, China), and DNA sequences were aligned with the complete CDS sequence of *Bos1* downloaded from the NCBI gene bank (accession number AF396827.2) to remove introns and locate the right open reading frame. Then, DNA sequences were translated into protein sequences, and the protein sequences of resistant isolates were aligned with sensitive ones to detect possible mutations.

### 2.6. Statistical Analysis

Data collected in this study were analyzed using IBM SPSS Statistics 19. Data collected from different isolates were used for ANOVA, after which post-hoc pairwise comparisons between mean values were performed using Fisher’s LSD method at *p* = 0.05.

## 3. Results

### 3.1. The Sensitivity of B. cinerea Isolates to Fludioxonil

**Figure 1:**
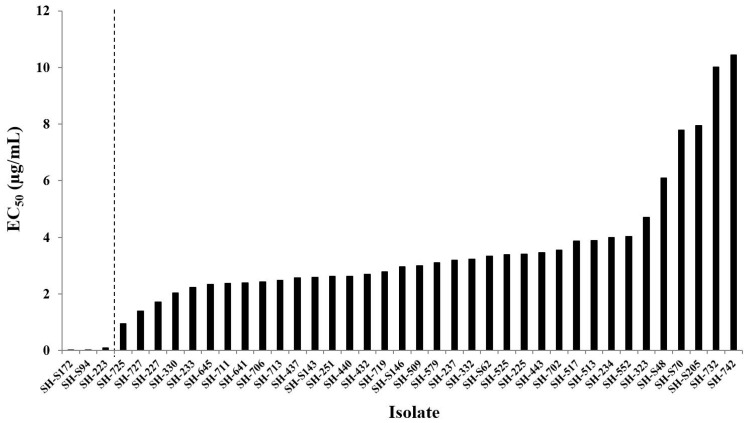
EC_50_ (effective concentration inhibiting 50% of colony growth) values of fludioxonil on mycelial growth of three sensitive isolates and 36 (highly resistant) HR isolates of *B. cinerea*. The HR isolates were first selected by discriminatory concentrations of fludioxonil, and the EC_50_ values were arranged incrementally. The isolates that were able to grow at 100 µg/mL fludioxonil were considered HR, being different from those moderately resistant (MR) isolates that could grow at 10 but not at 100 µg/mL. The columns on the left of the dashed line indicate the sensitive isolates, and those on the right of the dashed line indicate HR isolates.

A total of 187 field *B. cinerea* isolates from seven different districts of Shanghai province were used for a fludioxonil sensitivity test using discriminatory concentrations. They were categorized as sensitive, MR, or HR. Of the isolates, 65 (34.76%) were found to be resistant to fludioxonil, with 36 (19.25%) showing high resistance and 29 (15.51%) showing moderate resistance. To further detail the resistance level of the HR isolates, we determined the EC_50_ values of fludioxonil on the mycelial growth of the 36 HR isolates and three sensitive isolates (SH-S172, SH-S94, and SH-223). The results showed that the EC_50_ values for HR isolates ranged from 0.95 to 10.44 µg/mL, with a mean of 3.66 µg/mL. In contrast, the EC_50_ values for the sensitive isolate were only from 0.009 to 0.089 µg/mL, with a mean of 0.04 µg/mL (Figure 1). A previous study documented that the EC_50_ values of fludioxonil on the mycelial growth of *B. cinerea* for apple isolates were only from 0.003 to 0.038 µg/mL, with a mean of 0.005 µg/mL, and the EC_50_ values for pear isolates were 0.003 to 0.008 µg/mL, with a mean of 0.005 µg/mL [33]. These figures are in broad agreement with the EC_50_ values for the sensitive isolates used in the current research, which further demonstrated that the HR isolates in this study had a high resistance level.

### 3.2. The Sensitivity of HR Isolates to Other Fungicides

*B. cinerea* is at high risk to develop resistance to fungicides, and multiple resistance has been reported in this species [4,34,35]. To better understand multiple resistance combined with fludioxonil in the field *B. cinerea* isolates from Shanghai, the 31 resistant (19 HR and 12 MR) isolates were further exposed to other fungicides with different modes of action (Table 1). Regarding fludioxonil and the fungicides listed in Table 1, only one of these isolates (3.23%) was merely resistant to fludioxonil, while the other 30 of the 31 resistant isolates (96.77%) showed multiple resistance combined with fludioxonil, with three isolates (9.68%) showing resistance to two fungicides, eight (25.81%) showing resistance to three fungicides, 14 (45.16%) showing resistance to four fungicides, and five (16.13%) showing resistance to five fungicides simultaneously (Table 2). Notably, boscalid and fluopyram resistance often developed together, because these two fungicides have the same target protein. However, several isolates were only resistant to one of the two fungicides, which might have resulted from different resistance mechanisms.

It has been reported that the overexpression of the *atrB* gene can be observed in the fludioxonil-resistant isolates in *B. cinerea*, which could result in multidrug resistance (MDR) including cyprodinil [18,29]. To investigate whether the fludioxonil-resistant isolates obtained in this study also showed MDR phenotypes, a total of 23 isolates including 15 HR isolates (SH-225, SH-227, SH-234, SH-237, SH-251, SH-330, SH-332, SH-437, SH-641, SH-S62, SH-S143, SH-S205, SH-233, SH-440, and S146) and eight MR isolates (SH-309, SH-335, SH-338, SH-473, SH-458, SH-595, SH-631, and SH-S148) were used for cyprodinil sensitivity determination. The results showed that only two isolates (SH-S205 and SH-S148) were resistant to cyprodinil (Table 2), which indicated that most of the experimental isolates did not have MDR. Furthermore, the expression levels of the *atrB* gene in three fludioxonil-resistant isolates (SH-309, SH-641, and SH-458) and one representative sensitive isolate (SH-S172) were detected. It was found that the expression level of the *atrB* gene had no dramatic increase in the fludioxonil-resistant isolates (Figure S1). The lack of cross resistance between pyrimethanil and cyprodinil might also have resulted from different resistance mechanisms.

Moreover, these fludioxonil-resistant isolates are potentially also resistant to dicarboximide fungicides such as iprodione because they have similar modes of action targeting the same protein in a signaling pathway, and positive cross-resistance between fludioxonil and dicarboximide fungicides have been constantly noticed [22,36,37]. To test if the fludioxonil-resistant isolates were simultaneously resistant to dicarboximide fungicide, a total of 20 isolates including 12 HR isolates (SH-225, SH-227, SH-234, SH-237, SH-251, SH-330, SH-332, SH-437, SH-641, SH-S62, SH-S143, and S146) and eight MR isolates (SH-309, SH-335, SH-338, SH-473, SH-562, SH-595, SH-631, and SH-S148) were used for iprodione sensitivity determination. As a result, most of these fludioxonil-resistant isolates also showed resistance to iprodione, with the exceptions of three isolates (SH-437, SH-473, and SH-S143) (Table 2). Thus, multiple fungicide resistance of *B. cinerea* isolates is very common in Shanghai province, making it even more difficult to control this pathogen.

### 3.3. Biological Characteristics of Fludioxonil-Resistant Isolates

The development of fungicide resistance may influence the fitness of plant pathogens in the field, which could be reflected by different biological characteristics. Therefore, four fludioxonil-resistant isolates and three sensitive isolates were selected for fitness investigation. Among the selected resistant isolates, SH-S205 was resistant to two fungicides, SH-330 was resistant to four fungicides, SH-227 was resistant to five fungicides, and the other isolate (SH-309) was simultaneously resistant to six fungicides (Table 3). The three sensitive isolates were selected because they were sensitive to all of the tested fungicides. Different biological characteristics of the resistant group and sensitive group were compared, including mycelium growth rate, sporulation capacity, conidial germination, and pathogenicity. The results showed that the mycelium growth rate was relatively consistent among different sensitive isolates, and only the six-fungicide-resistant isolate displayed a slower mycelium growth, with other resistant isolates being comparable or even faster than the sensitive group. The asexual reproduction of the resistant isolates was weakened to varying degrees, given that their sporulation amount was much lower than sensitive ones. The conidial germination rate of the resistant group was also significantly lower than that of the sensitive group. As for pathogenicity on tomatoes, all of the isolates could infect tomato fruits and cause lesions, and no distinct difference was observed between the sensitive and resistant groups, except for the six-fungicide-resistant isolate, which caused significantly smaller lesions on tomato fruits (Table 3). Considering all the tested factors, the fitness of resistant isolates was a little lower than that of sensitive ones, but they could still be greatly competitive in the field, which may develop into a substantial concern in agricultural production.

### 3.4. Osmotic Sensitivity of B. cinerea Isolates to Fludioxonil

**Figure 2:**
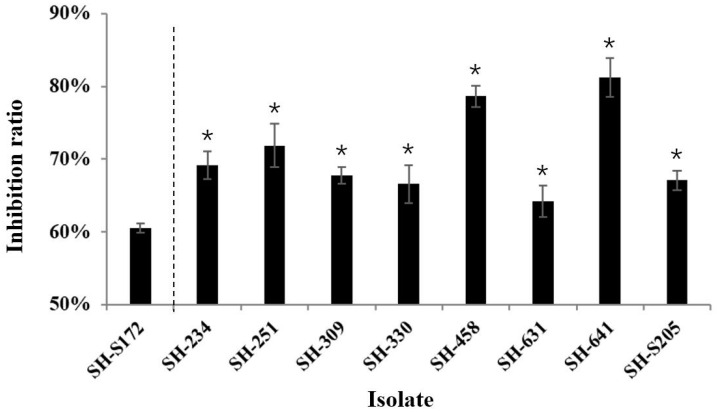
Osmotic sensitivity comparation between fludioxonil-resistant isolates and one representative sensitive isolate. The inhibition ratio of NaCl (40 g/L) on the mycelium growth of different isolates was determined. Values represent the mean ± SD of three replicates, and the asterisk above column means that the inhibition ratio of the resistant isolate is significantly different from that of the sensitive one. * *p* < 0.05.

It has been frequently reported that fludioxonil-resistant isolates become less tolerant to osmotic stress in different species [12,14,16,22]. To learn about the osmotic sensitivity of the fludioxonil-resistant isolates from Shanghai province, the tolerance to NaCl under a high concentration (40 g/L) was compared between eight resistant isolates (SH-234, SH-251, SH-309, SH-330, SH-458, SH-631, SH-641, and SH-S205) and one representative isolate (SH-S172). It turned out that the inhibition ratio of NaCl on the mycelium growth of fludioxonil-resistant isolates was significantly higher than that of the sensitive isolate (Figure 2), indicating that the fludioxonil-resistant isolates were more sensitive to osmotic stress. It could also be noticed that the tolerance of fludioxonil-resistant isolates was varying, with some isolates being very close to the sensitive one, which might be a reason explaining the large population of resistant isolates present in the field.

### 3.5. Molecular Resistance Mechanisms of B. cinerea Isolates to Fludioxonil

**Figure 3:**
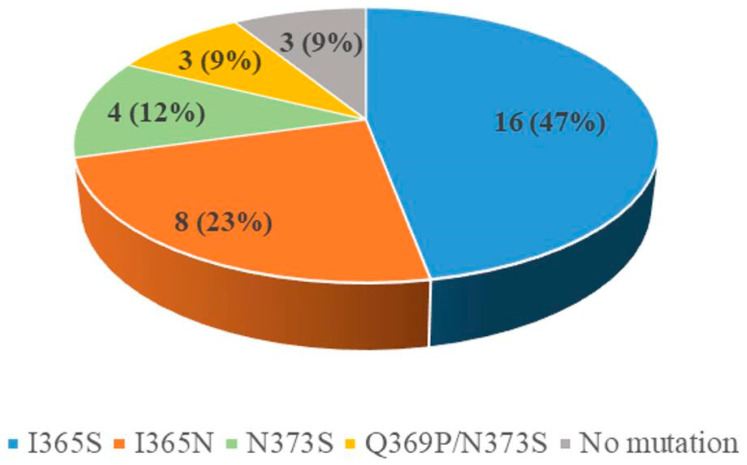
Mutation types of the Bos1 protein and their proportions in the resistant group. The figure in the pie chart block indicates the number of isolates with each mutation type, with the proportion being shown in parentheses.

Though the mode of action of fludioxonil on pathogens is still not completely clear, the protein histidine kinase Os1 is probably the primary target of this fungicide, and a series of mutations conferring fludioxonil resistance has been identified in Os1 [8,16,22,38,39]. Therefore, the *Bos1* gene was amplified from 34 fludioxonil-resistant isolates (22 HR and 12 MR) and three sensitive isolates, and the sequences of the protein products were compared to each other. The results showed that no resistance-related mutation was present in the protein of the three sensitive isolates, while different point mutations were identified in the most of the resistant isolates, including I365S (from isoleucine to serine at the 365th amino acid) in isolates SH-641, SH-702, SH-631, SH-728, SH-225, SH-237, SH-251, SH-458, SH-323, SH-332, SH-595, SH-562, SH-335, SH-338, SH-437, and SH-440; I365N (from isoleucine to asparagine at the 365th amino acid) in isolates SH-719, SH-S146, SH-S148, SH-S205, SH-473, SH-227, SH-233, and SH-234; N373S (from asparagine to serine at the 373rd amino acid) in isolates SH-714, SH-725, SH-S62, and SH-S143; and combined mutations of Q369P (from glutamine to proline at the 369th amino acid) and N373S (Q369P/N373S) in isolates SH-330, SH-517, and SH-537 (Figure 3 and Figure S2). These point mutations have been found in fludioxonil- and iprodione-resistant isolates of *B. cinerea* in previous studies [8,16,22,39,40,41], further confirming that these two fungicides have a similar mode of action. It seemed that mutations of the isoleucine at the position of 365 tended to occur, since most of the resistant isolates contained such amino substitutions (either I365S or I365N). In several fludioxonil-resistant isolates (isolates 309, 432, and 727), no mutations were identified (Figure 3), which may have been due to other resistance mechanisms. Overall, the point mutations of the Bos1 protein might be mainly responsible for field fungicide resistance of *B. cinerea* to fludioxonil and iprodione in Shanghai province.

## 4. Discussion

Fungicide application is the most simple and effective method for the control of plant diseases caused by fungal pathogens. However, fungicide resistance development in the field has become a serious problem in agricultural production. Fludioxonil is a fungicide that inhibits a variety of fungal pathogens by interfering with the HOG1 cascade of the MAPK signaling pathway [8,22]. Even though the mode of action of fludioxonil has not been fully described, the fact that a range of mutations in the os1 protein confer resistance to different fungal pathogens to this compound makes it plausible that fludioxonil primarily targets this protein [14,15,16]. The gray mold causal agent *B. cinerea* is economically important and has traits to develop resistance to different fungicides, and fludioxonil has become an important fungicide for the control of this disease [7,8]. Clarifying the field fungicide resistance of *B. cinerea* to fludioxonil, as well as other fungicides, would give more scientific reference for the instruction of fungicide application.

Though laboratory mutants of *B. cinerea* HR to fludioxonil are frequently reported, notably, only a low level of fludioxonil resistance was detected in field populations [22]. *B. cinerea* isolates HR to fludioxonil in the field had been reported from different provinces in China including Shandong and Sichuan, but very few HR isolates were found [16,39]. This is likely because the strains with high levels of resistance are hypersensitive to osmotic stress, and they usually display reduced pathogenicity to hosts, making them much less competitive than those sensitive ones in the field [28,42,43]. The appearance of *B. cinerea* isolates HR to fludioxonil is worrying, and a deeper analysis concerning the resistance mechanisms is urgently required. In this study, we found that the field resistance of *B. cinerea* to fludioxonil was much popular in Shanghai province, with 19.25% and 15.51% of isolates showing high and moderate resistance, respectively. The difference in resistance frequencies probably resulted from the disease occurrence frequency and fungicide application history. Most of the tested fludioxonil-resistant isolates were resistant not only to fludioxonil but also to some other fungicides, including carbendazim, boscalid, fluopyram, azoxystrobin, pyrimethanil, difenoconazole, and iprodione, indicating that cross resistance has developed in this region. This phenomenon is worrying because it means fewer fungicides are effective against these isolates. The other fungicides used in this study have different modes of action with fludioxonil except for iprodione, so it is less likely that the cross resistance was from target-based or pathway-based cross-resistance [44]. However, the resistance mechanisms of these isolates to other fungicides need to be further verified.

Biological characteristics and osmatic sensitivity investigations showed that the resistant isolates had a lower fitness compared to sensitive ones, especially in asexual production and conidial germination, but they could still be greatly competitive given that their growth and pathogenicity did not reduce dramatically, which may explain why the proportion of resistant isolates in the field was relatively high. Only four resistant isolates were used for biological characteristics investigation in this study. Even though they showed similar trend in fitness compared to sensitive ones, it is plausible that other resistant isolates have different traits. Hopefully, the proportion of resistant isolates in the field could be reduced after a certain period of time without the application of fludioxonil or dicarboximide fungicides, given that the fitness of resistant isolates was lower.

Comparing the sequences of Bos1 from sensitive and resistant isolates identified different mutations in this protein that have been reported in fludioxonil- and iprodione-resistant isolates of *B. cinerea* in previous studies [8,16,22,39,40,41]. Even though point mutations in Bos1 have been frequently reported in fludioxonil-resistant isolates of *B. cinerea* [8,16,22,39], the relationship between mutations and resistance still needs clarification. Furthermore, the same point mutations in Bos1 can be found in different fludioxonil resistance phenotypes [40,41]. Other possible resistance mechanisms include mutations in Os-like or Os-related proteins, the overexpression of the target protein or target-related proteins, and the overexpression of ABC transporters and major facilitator superfamily transporters [18,19,45,46,47]. Therefore, the point mutations in Bos1 that were identified in this study might contribute to fludioxonil and iprodione resistance, but it cannot be excluded that other mechanisms also play a part in the resistance of *B. cinerea*. The mutations in the target protein are heritable and fungicide resistance resulting from the target mutation is usually stable, so there is an urgent need to apply new management methods to gray mold disease, including breeding disease-resistant varieties and developing novel fungicides. In a few fludioxonil-resistant isolates, no point mutations were found in the potential target protein Bos1, which is not surprising because previous studies have also reported some *B. cinerea* isolates without mutations in this protein showing resistance to this fungicide [16,22]. It is more likely that these resistant isolates without mutations might adopt other fungicide resistance mechanisms mentioned earlier. Taken together, the results show that the field fludioxonil resistance of *B. cinerea* in Shanghai province is relatively high, and the Bos1 amino acid sequence mutation might be an important basis for resistance developing in the population.

## Figures and Tables

**Table 1:** Other fungicides with different modes of action used in this study for sensitivity tests with fludioxonil-resistant isolates.

Chemical Class	Fungicide	Mode of Action ^a^
Benzimidazoles	Carbendazim	Inhibition of β-tubuline assembly in mitosis
Pyridine-carboxamides	Boscalid	Inhibition of complex II: succinate-dehydrogenase in respiration
Pyridinyl-ethyl-benzamides	Fluopyram	Inhibition of complex II: succinate-dehydrogenase in respiration
Strobilurins	Azoxystrobin	Inhibition of complex III: cytochrome bc1 (ubiquinol oxidase) at quinone-outside (Qo) site in respiration
Anilinopyrimidines	Pyrimethanil	Inhibition of methionine biosynthesis (proposed)
Triazoles	Difenoconazole	Inhibition of C14-demethylase in sterol biosynthesis

^a^ The mode of action of each fungicide is quoted from the Fungicide Resistance Action Committee (www.frac.info).

**Table 2:** The resistance of fludioxonil-resistant isolates to other fungicides.

Isolate		Resistance to Fungicides ^a^								Location
	Flud	Car	Azox	Bos	Fluop	Dif	Pyr	Cyp	Ipr	
SH-S205	HR	S	S	S	S	S	S	R	na	Chongming
SH-537	MR	S	S	S	S	R	S	na	na	Fengxian
SH-225	HR	S	S	S	S	S	R	S	R	Jinshan
SH-562	MR	S	S	S	S	S	R	na	R	Fengxian
SH-458	MR	S	S	R	S	S	R	S	na	Fengxian
SH-338	MR	S	S	R	S	S	R	S	R	Jinshan
SH-251	HR	S	R	S	S	S	R	S	R	Jinshan
SH-234	HR	S	R	S	S	S	R	S	R	Jinshan
SH-330	HR	S	S	S	S	R	R	S	R	Jinshan
SH-595	MR	S	S	S	S	R	R	S	R	Songjiang
SH-719	HR	R	S	S	S	S	R	na	na	Fengxian
SH-725	HR	R	S	S	S	S	R	na	na	Fengxian
SH-728	MR	R	R	S	R	S	S	na	na	Pudong New Area
SH-233	HR	S	R	R	R	S	S	S	na	Jinshan
SH-473	MR	R	S	R	S	S	R	S	S	Fengxian
SH-227	HR	R	S	R	S	S	R	S	R	Jinshan
SH-332	HR	S	S	R	S	R	R	S	R	Jinshan
SH-237	HR	S	R	R	S	S	R	S	R	Jinshan
SH-440	HR	S	R	R	S	S	R	S	na	Fengxian
SH-437	HR	S	S	R	R	S	R	S	S	Fengxian
SH-631	MR	S	S	R	R	S	R	S	R	Songjiang
SH-714	MR	R	R	S	S	S	R	na	na	Jinshan
SH-702	HR	R	R	S	S	S	R	na	na	Pudong New Area
SH-S143	HR	R	S	S	R	S	R	S	S	Chongming
SH-S146	HR	R	S	S	R	S	R	S	R	Chongming
SH-335	MR	S	R	R	R	S	S	S	R	Jinshan
SH-309	MR	S	R	R	R	S	R	S	R	Jinshan
SH-727	HR	R	R	S	S	R	R	na	na	Pudong New Area
SH-S148	MR	R	S	R	R	S	R	R	R	Chongming
SH-S62	HR	R	R	S	R	S	R	S	R	Pudong New Area
SH-641	HR	S	S	R	R	R	R	S	R	Songjiang

^a^ S: sensitive to specific fungicide; R: resistant to specific fungicide; HR: highly resistant to fludioxonil; MR: moderately resistant to fludioxonil; Flud: fludioxonil; Car: carbendazim; Azox: azoxystrobin; Bos: boscalid; Fluop: fluopyram; Dif: difenoconazole; Pyr: pyrimethanil; Cyp: cyprodinil; Ipr: iprodione; na: not analyzed.

**Table 3:** Biological characteristics of fludioxonil-resistant isolates and sensitive isolates.

Isolate		Resistance to Fungicides								Colony Diameter (mm)	Sporulation (cm^−2^)	Germination (%)	Lesion Area (mm^2^)	CFI (×10^7^)
	Flud	Car	Azox	Bos	Fluop	Dif	Pyr	Cyp	Ipr					
SH-223	S	S	S	S	S	S	S	na	na	23.17 cd	144.17 a	91.67 a	775.67 a	23.75
SH-S94	S	S	S	S	S	S	S	na	na	24.17 c	130.17 b	97.33 a	575.33 c	17.62
SH-S172	S	S	S	S	S	S	S	na	na	23.33 c	148.50 a	92.33 a	509.00 d	16.28
SH-S205	HR	S	S	S	S	S	S	R	na	26.33 b	77.00 cd	74.67 b	727.67 b	11.02
SH-330	HR	S	S	S	S	R	R	S	R	22.17 d	34.50 e	75.67 b	527.33 d	3.05
SH-227	HR	R	S	R	S	S	R	S	R	29.00 a	85.33 c	69.67 b	778.00 a	13.41
SH-309	MR	S	R	R	R	S	R	S	R	21.00 e	68.00 d	73.33 b	455.33 e	4.77

^a^ S: sensitive to specific fungicide; R: resistant to specific fungicide; HR: highly resistant to fludioxonil; MR: moderately resistant to fludioxonil; Flud: fludioxonil; Car: carbendazim; Azox: azoxystrobin; Bos: boscalid; Fluop: fluopyram; Dif: difenoconazole; Pyr: Pyrimethanil; Cyp: cyprodinil; Ipr: iprodione; na: not analyzed. ^b^ CFI: combined fitness index, calculated as in vitro colonial diameter × sporulation × conidial germination × lesion area. Values in a column followed by the same letter are not significantly different (*p* < 0.05).

## Supplementary Materials

The following are available online at https://www.mdpi.com/2076-2607/9/2/266/s1: Figure S1: Relative expression of the *atrB* gene in three fludioxonil-resistant isolates and one representative sensitive isolate; Figure S2: Alignment of Bos1 protein sequences from different *Botrytis cinerea* isolates; Table S1: Fungicides used in this study; Table S2: Sensitivity test of different fungicides.
