# Supplementary material for: Characterization of the Field Fludioxonil Resistance and Its Molecular Basis in Botrytis cinerea from Shanghai Province in China
Source: Microorganisms. 2021 Jan 28;9(2):266. doi: 10.3390/microorganisms9020266 (PMC7912569; doi:10.3390/microorganisms9020266)
Supplement: Supplementary file 1 [file microorganisms-09-00266-s001.pdf]

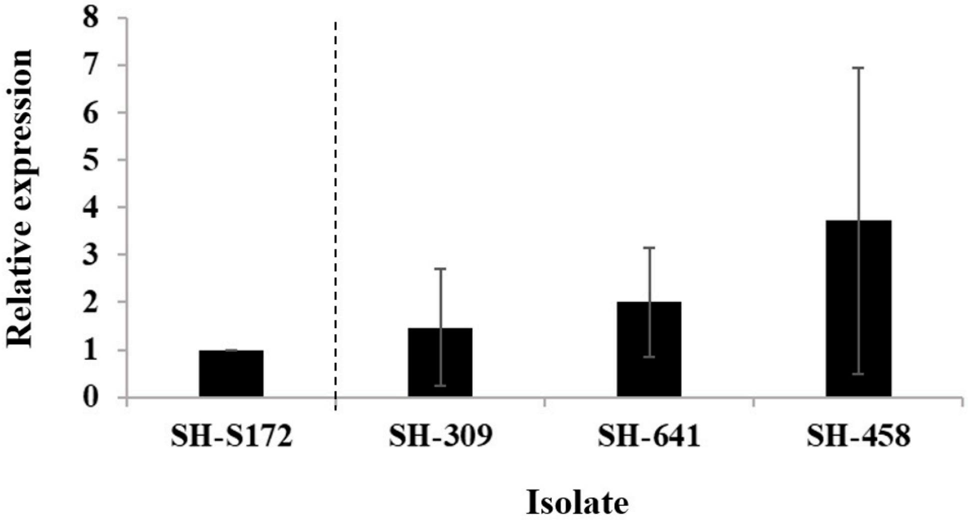

Figure S1. Relative expression of the *atrB* gene in three fludioxonil-resistant isolates and one representative sensitive isolate. The transcript levels of the *atrB* gene in fludioxonil-resistant isolates are shown relative to the sensitive one (SH-S172), which was assigned a value of 1.0. Values represent the mean ± SD of two biological replicates.

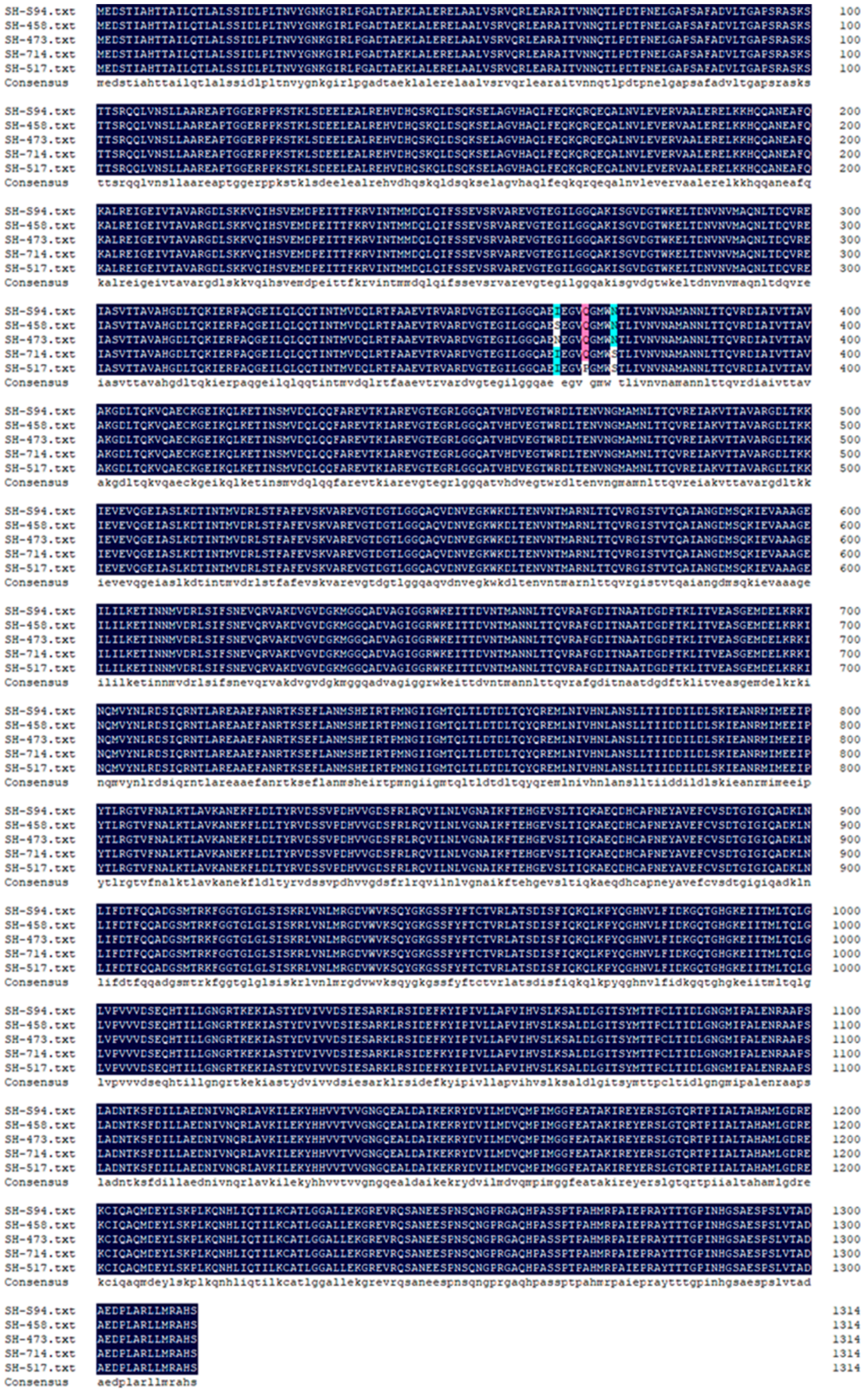

Figure S2. Alignment of Bos1 protein sequences from different *Botrytis cinerea* isolates. Each type of protein sequence is indicated by one representative isolate. SH-94: without resistance related mutations, SH-458: with I365S mutation, SH-473: with I365N mutation, SH-714: with N373S mutation, SH-517: with Q369P/N373S mutations.

Table S1. Fungicides used in this study.

Fungicide	Active ingredient (a.i.)	Provider
fludioxonil	95%	Syngenta Biotechnology Co., Ltd., Shanghai, China
azoxystrobin	98%	Syngenta Biotechnology Co., Ltd., Shanghai, China
pyrimethanil	95.2%	Syngenta Biotechnology Co., Ltd., Shanghai, China
carbendazim	98%	Guangxin Agricultural Chemicals Co., Ltd, Anhui, China.
boscalid	95%	Guangxin Agricultural Chemicals Co., Ltd., Anhui, China.
fluopyram	98.8%	Guangxin Agricultural Chemicals Co., Ltd., Anhui, China.
difenoconazole	95%	Yulong Chemical Industrial Co., Ltd., Hangzhou, China.
cyprodinil	98.5%	Beijing Mindleader Agroscience Co.,Ltd., Beijing, China
iprodione	95.5%	Bayer CropScience Co., Ltd., Beijing, China.

Table S2. Sensitivity test of different fungicides.

Fungicide	Medium	Discrimination method [a]	Discriminatory concentration (μg/mL)	Reference
fludioxonil	PDA	MIC	10, 100	22
carbendazim	YG	MIC	100	23
azoxystrobin	YBA	MIC	100	24
boscalid	YBA	MIC	10	25
fluopyram	YBA	MIC	10	26
difenoconazole	YG	EC_{50}	2.68	Unpublished data
pyrimethanil	GKMN	MIC	1	25, 27
cyprodinil	GKMN	EC_{50}	10	28
iprodione	PDA	EC_{50}	1	28

[a] MIC means the minimum inhibitory concentration, and EC_{50} indicates the effective concentration inhibiting 50% of colony growth.
